# Different Localization Patterns of Anthocyanin Species in the Pericarp of Black Rice Revealed by Imaging Mass Spectrometry

**DOI:** 10.1371/journal.pone.0031285

**Published:** 2012-02-17

**Authors:** Yukihiro Yoshimura, Nobuhiro Zaima, Tatsuya Moriyama, Yukio Kawamura

**Affiliations:** Department of Applied Biological Chemistry, Graduate School of Agriculture, Kinki University, Nara, Japan; Max Planck Institute for Chemical Ecology, Germany

## Abstract

Black rice (*Oryza sativa* L. *Japonica*) contains high levels of anthocyanins in the pericarp and is considered an effective health-promoting food. Several studies have identified the molecular species of anthocyanins in black rice, but information about the localization of each anthocyanin species is limited because methodologies for investigating the localization such as determining specific antibodies to anthocyanin, have not yet been developed Matrix-assisted laser desorption/ionization imaging mass spectrometry (MALDI-IMS) is a suitable tool for investigating the localization of metabolites. In this study, we identified 7 species of anthocyanin monoglycosides and 2 species of anthocyanin diglycosides in crude extracts from black rice by matrix-assisted laser desorption/ionization mass spectrometry (MALDI-MS) analysis. We also analyzed black rice sections by MALDI-IMS and found 2 additional species of anthocyanin pentosides and revealed different localization patterns of anthocyanin species composed of different sugar moieties. Anthocyanin species composed of a pentose moiety (cyanidin-3-*O*-pentoside and petunidin-3-*O*-pentoside) were localized in the entire pericarp, whereas anthocyanin species composed of a hexose moiety (cyanidin-3-*O*-hexoside and peonidin-3-*O*-hexoside) were focally localized in the dorsal pericarp. These results indicate that anthocyanin species composed of different sugar moieties exhibit different localization patterns in the pericarp of black rice. This is the first detailed investigation into the localization of molecular species of anthocyanins by MALDI-IMS.

## Introduction

Rice (*Oryza sativa* L.) is one of the most important cereal crops in the world, especially in Asian countries. One of the various rice cultivars is black rice, which contains high levels of anthocyanins in the pericarp and is considered an effective health-promoting food. The crude anthocyanin-rich extracts of black rice bran improve serum triglyceride levels, which contributes to the suppression of atherosclerosis [1], [2], and protects against insulin resistance [3], alcoholic liver injury [4], and light-induced retinal damage [5], [6]. Due to these human health benefits, anthocyanin compositions have been investigated not only in black rice but also in various plants, such as berries [7], black soybeans [8], [9], [10], and dark-colored grapes [11], [12]. Several studies of the molecular species of anthocyanins in black rice have identified cyanidin-3-*O*-glucoside, cyanidin-3-*O*-galactoside, peonidin-3-*O*-glucoside, and cyanidin-3-*O*-rutinoside [13], [14], [15], [16]. However, there are virtually no localization studies of individual anthocyanin species in plant tissue because methodologies for investigating the localization, such as determining specific antibodies to anthocyanin, have not yet been developed. Matrix-assisted laser desorption/ionization imaging mass spectrometry (MALDI-IMS) is an emerging technology that allows the simultaneous investigation of the content and spatial distribution of a wide range of biomolecules such as lipids [17], [18], [19], glycolipids [20], [21], amino acids [22], proteins [23], and peptides [24], [25], as well as administered pharmaceuticals [26] without requiring antibodies, staining, or complicated pretreatment steps. Our previous study demonstrated that MALDI-IMS combined with the Kawamoto method, which uses adhesive films to prepare the cryosections, is an effective method for investigating the localization and composition of rice metabolites [27]. In this study, we investigated the localization of several anthocyanin species in black rice tissue in detail by MALDI-IMS and revealed the different localization patterns of anthocyanin species in the pericarp of black rice.

## Results

### Identification of anthocyanins in the crude extract from black rice seed

**Figure 1:**
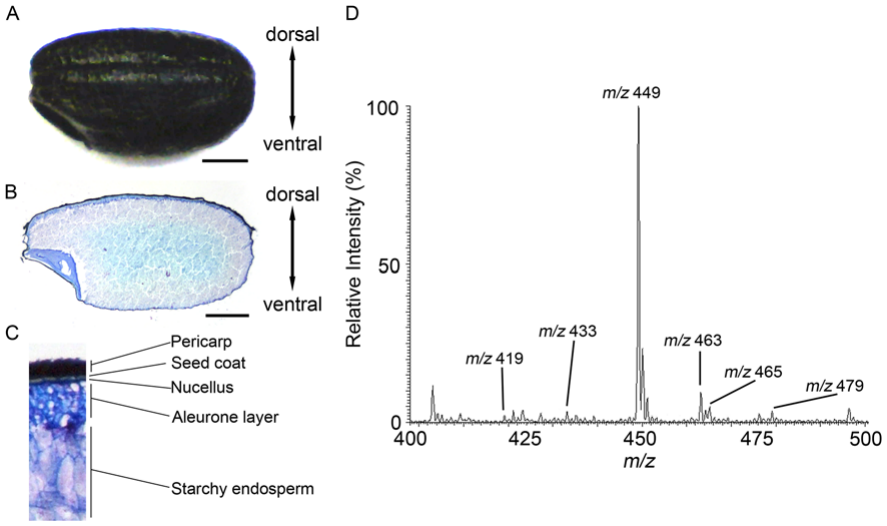
Identification of anthocyanin species in black rice crude extract. A, Appearance of black rice. B, Black rice section stained with toluidine blue O. Scale bar: 1.0 mm. C, Enlarged image of the dorsal region of the longitudinal black rice section. D, Mass spectrum of the black rice crude extract.

**Figure 2:**
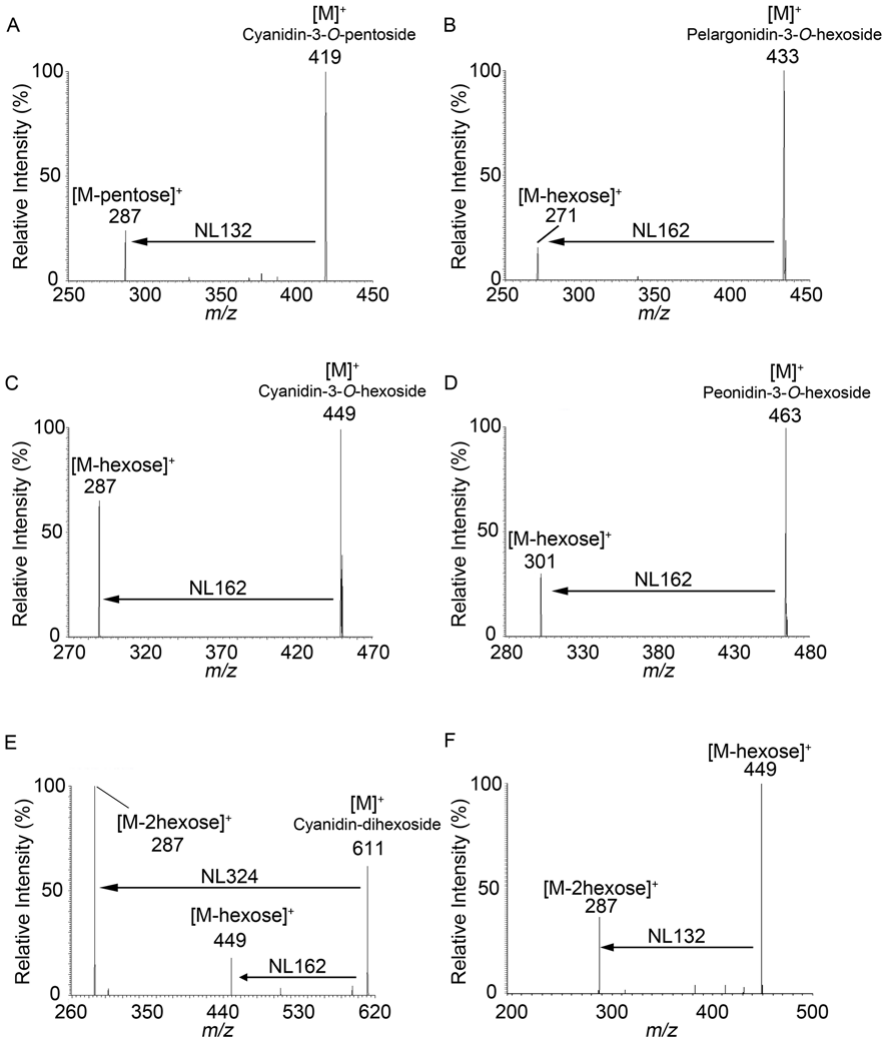
Representative MS/MS spectra of ions in black rice crude extract. A–D, [M]^+^ ions at *m/z* 419 (A), *m/z* 433 (B), *m/z* 449 (C), and *m/z* 463 (D). Neutral losses (NL) of 132 Da and 162 Da indicate losses of a pentose and hexose, respectively. E, F, [M]^+^ ions at *m/z* 611 (E) and MS^3^ spectrum of ions at *m/z* 449 derived from ions at *m/z* 611 (F).

Figure 1A is an image of a whole black rice seed used in this study. A 10-µm- thick cryosection shows that the black or purple pigment is localized in the pericarp and seed coat layers (Fig. 1B, C) and that the pericarp and aleurone layers were thicker in the dorsal side than in the ventral side (Fig. 1B). We used MALDI-MS to analyze the crude extract to identify the anthocyanins in the pigmented layers of black rice. As shown in Figure 1D, abundant anthocyanin signals at *m/z* 419, 433, 449, 463, 465, and 479 were observed in the mass spectrum. We analyzed precursor ions by tandem mass spectrometry (MS/MS) to identify the molecular species of anthocyanins in the crude extract. The MS/MS spectrum of the *m/z* 419 ion in the crude extract demonstrated an intense ion at *m/z* 287 (neutral loss of 132 Da), which would correspond to the loss of a pentose moiety (Fig. 2A). Unfortunately, the MALDI-MS system could not differentiate between molecular species of sugar moieties that have the same molecular mass, such as xylose and arabinose. The MS/MS fragmentation of the ion at *m/z* 287 would correspond to the cyanidin aglycone. Thus, we identified the *m/z* 419 ion as an [M]^+^ ion of cyanidin-3-*O*-pentoside (Fig. 2A). The MS/MS spectrum of the *m/z* 433 ion demonstrated an ion at *m/z* 271 (neutral loss of 162 Da), which would correspond to the loss of a hexose moiety (glucose or galactose), and the product ion at *m/z* 271 ion would correspond to the pelargonidin aglycone (Fig. 2B). Therefore, we identified the *m/z* 433 ion as an [M]^+^ ion of pelargonidin-3-*O*-hexoside (Fig. 2B). Similarly, because the ions in the black rice crude extract at *m/z* 449, 463, 465, and 479 also yielded the characteristic neutral loss of 162 Da corresponding to the loss of a hexose moiety, these ions were identified as the [M]^+^ ions of cyanidin-3-*O*-hexoside (Fig. 2C), peonidin-3-*O*-hexoside (Fig. 2D), delphinidin-3-*O*-hexoside, and petunidin-3-*O*-hexoside (Table 1), respectively. The molecular species identified in MS/MS analyses are summarized in Table 1. Recently, cyanidin-3-*O*-gentiobioside consisting of 2 glucose moieties and a cyanidin aglycone was identified in black rice [28]. We performed MS/MS analyses on the *m/z* 611 and 625 precursor ions to examine the possibility that this anthocyanin diglycoside might be included in the black rice crude extract used in this study. MS/MS analysis of the precursor ions at *m/z* 611 yielded an ion at *m/z* 287 corresponding to the aglycone cyanidin, which could be formed by a neutral loss of 2 hexoses (Fig. 2E). A small peak detected at *m/z* 449 indicated the possibility that the ions formed with the loss of a hexose moiety (Fig. 2E). To examine whether the neutral loss of 324 Da (*m/z* 611→287) corresponded to the loss of 2 hexoses, we performed further MS^3^ analysis on the product ions at *m/z* 449 formed from the ions at *m/z* 611. The ions at *m/z* 287 were detected and were shown to be formed from the ions at *m/z* 449 with a neutral loss of 162 Da, corresponding to 1 hexose (Fig. 2F). These results indicate that the ions at *m/z* 287 were formed from the ions at *m/z* 611 with a loss of 2 hexoses. Thus, we confirmed that the ions at *m/z* 611 in the black rice crude extract were [M]^+^ ions of cyanidin dihexoside.

**Table 1:** Identified anthocyanin species in this study.

Parent ion [M]^+^ *m/z*	Fragment ions [M]^+^ *m/z*	Identified anthocyanin
**419**	287	Cyanidin-3-*O*-pentoside
**433**	271	Pelargonidin-3-*O*-hexoside
**449**	287	Cyanidin-3-*O*-hexoside
**449**	317*	Petunidin-3-*O*-pentoside*
**463**	301	Peonidin-3-*O*-hexoside
**463**	331*	Malvidin-3-*O*-pentoside*
**465**	303	Delphinidin-3-*O*-hexoside
**479**	317	Petunidin-3-*O*-hexoside
**493**	331	Malvidin-3-*O*-hexoside
**611**	287/449	Cyanidin-dihexoside
**625**	301/463	Peonidin-dihexoside

*Identified only by MALDI-IMS/MS on black rice tissue.

Similarly, we identify [M]^+^ ions at *m/z* 625 as another anthocyanin dihexoside, which consisted of 2 hexoses and peonidin as an aglycone (Table 1).

### IMS analysis of anthocyanins in black rice tissue

**Figure 3:**
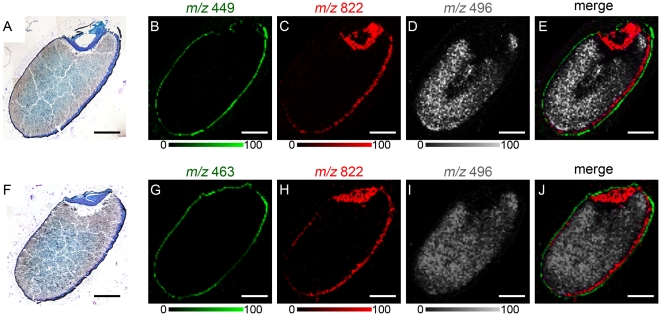
MALDI-IMS analysis of anthocyanins in black rice sections. A, Black rice section stained with toluidine blue O after MALDI-IMS analysis of the ions at *m/z* 449. B, Localization pattern of the ions at *m/z* 449. C, Localization pattern of the ions at *m/z* 822 corresponding to PC (diacyl C36:3), which marks the nucellus epidermis/aleurone layer. D, Localization pattern of the ions at *m/z* 496 corresponding to LPC (C16:0), which marks the endosperm. E, Merged ion image of *m/z* 449 (red), *m/z* 822 (green) and *m/z* 496 (white). F, Black rice section stained with toluidine blue O after MALDI-IMS analysis of the ions at *m/z* 463. G, Localization pattern of the ions at *m/z* 463. H, Localization pattern of the ions at *m/z* 822 corresponding to PC (diacyl C36:3). I, Localization pattern of the ions at *m/z* 496 corresponding to LPC (C16:0). J, Merged ion image of *m/z* 463 (red), *m/z* 822 (green) and *m/z* 496 (white). Scale bar: 1.0 mm.

We analyzed black rice sections by MALDI-IMS to investigate whether anthocyanins can be visualized in black rice tissue. As expected from the localization of black pigment in the sections (Figs. 1B, 3A), ions at *m/z* 449 predicted as cyanidin-3-*O*-hexoside were distributed in the outer pericarp and seed coat layers (Fig. 3B), and were clearly separated from the nucellus epidermis/aleurone layer marked by phosphatidylcholine (PC; diacyl C36:3) (Fig. 3C), and the endosperm was marked by lysophosphatidylcholine (LPC; C16:0) (Fig. 3D). Similarly, ions at *m/z* 463 predicted as peonidin-3-*O*-hexoside also were distributed in the outer pericarp and seed coat layers (Fig. 3G). Merged images of anthocyanins, PC, and LPC are shown in Figure 3E and J.

### IMS based on MS/MS analysis of anthocyanins on black rice tissue

**Figure 4:**
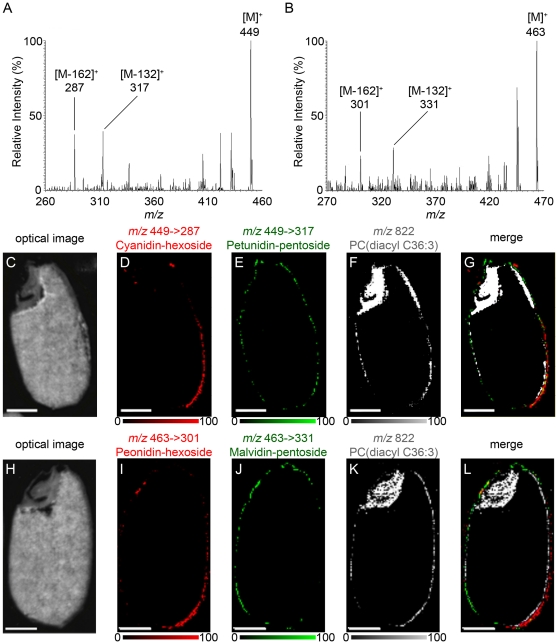
MALDI-IMS/MS analysis of the ions at *m/z* 449 and *m/z* 463. A, B, MS/MS spectrum of ions at *m/z* 449 (A) and *m/z* 463 (B) obtained from the pericarp of a black rice section. C, Optical image of a black rice section after analysis of the ions at *m/z* 449. D, E, Localization patterns of the fragment ions at *m/z* 287 (E) and 317 (F), derived from the parent ions at *m/z* 449, corresponding to cyanidin-3-*O*-hexoside and petunidin-3-*O*-pentoside, respectively. F, Localization pattern of the ions at *m/z* 822 corresponding to PC (diacyl C36:3), which marks the nucellus epidermis/aleurone layer. G, Merged ion image of *m/z* 449→287 (red), *m/z* 449→317 (green) and *m/z* 822 (blue). H, Optical image of a black rice section after analysis of the ions at *m/z* 463. I, J, Localization patterns of the fragment ions at *m/z* 301 (I) and *m/z* 331 (J), derived from parent ions at *m/z* 463, corresponding to peonidin-3-*O*-hexoside and malvidin-3-*O*-pentoside, respectively. K, Localization pattern of the ions at *m/z* 822 corresponding to PC (diacyl C36:3). L, Merged ion image of *m/z* 463→301 (red), *m/z* 463→331 (green), and *m/z* 822 (blue). Scale bar: 1.0 mm.

To confirm the molecular species of anthocyanins in black rice tissue, we analyzed the black rice section with MALDI-IMS based on MS/MS (MALDI-IMS/MS) (Fig. 4A, B). Peaks corresponding to a neutral loss of 162 Da, hexose, were detected at each MS/MS spectrum. The product ions at *m/z* 287 (neutral loss of 162 Da) derived from the precursor ions at *m/z* 449 correspond to the cyanidin aglycone (Fig. 4A), and the product ions at *m/z* 301 derived from *m/z* 463 correspond to the peonidin aglycone (Fig. 4B). Although a single product ion at *m/z* 287 corresponding to a neutral loss of 162 Da was detected at *m/z* 449 in MS/MS analysis of the crude extract (Fig. 2C), other product ions at *m/z* 317 were detected in MS/MS analysis of the black rice section (Fig. 4A). The product ion at *m/z* 317 derived from the precursor ions at *m/z* 449 resulted from the neutral loss of 132 Da, pentose, and corresponds to the petunidin aglycone. Similarly, both *m/z* 301 and *m/z* 331 ions were detected from *m/z* 463 precursor ions in MS/MS analysis of the tissue section (Fig. 4B). The product ion at *m/z 3*31 corresponds to the malvidin aglycone. These product ion images were used to investigate the distribution of each anthocyanin species in black rice tissue. Cyanidin-3-*O*-hexoside (*m/z* 449→287) was localized primarily in the dorsal posterior side of the pericarp (Fig. 4D, lower right corner), whereas petunidin-3-*O*-pentoside (*m/z* 449→317) was localized in the entire pericarp (Fig. 4E). Their distributions were different from the distribution of PC (diacyl C36:3), which was localized in the nucellus epidermis/aleurone layer (Fig. 4F). The merged image of these molecules is presented in Figure 4G. Similarly, peonidin-3-*O*-hexoside (*m/z* 463→301) was localized primarily in the dorsal posterior side of the pericarp (Fig. 4I, lower right corner), and malvidin-3-*O*-pentoside (*m/z* 463→331) was localized in the entire pericarp (Fig. 4J). We confirmed that their distributions were different from the distribution of PC (diacyl C36:3) (Fig. 4K, L). These results suggest that anthocyanins composed of pentose were distributed in the entire pericarp and anthocyanins composed of hexose were localized predominantly in the posterior dorsal side of the pericarp; in other words, the different localization patterns of anthocyanin species were due to different compositions of sugar moieties rather than to aglycones.

### Inter pericarp distribution of anthocyanins

**Figure 5:**
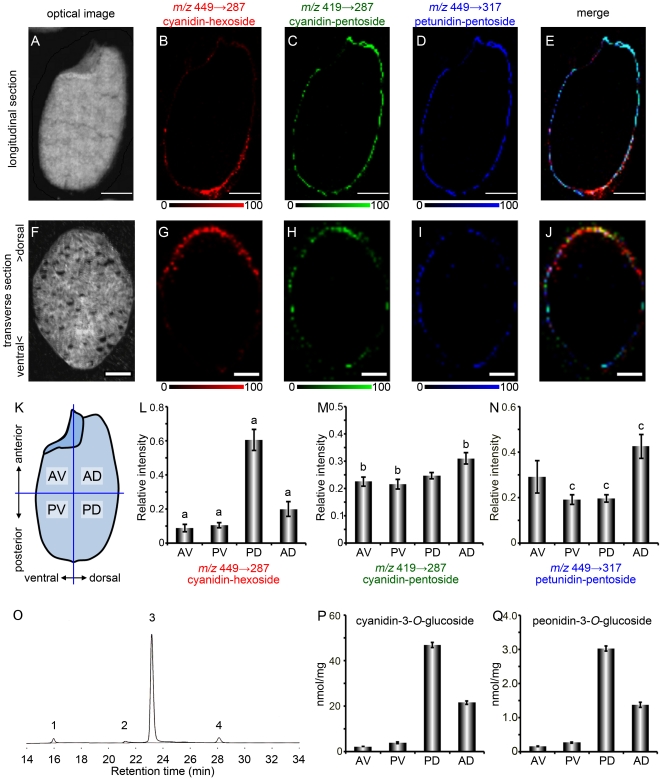
MALDI-IMS/MS analysis of ions at *m/z* 419 and *m/z* 449 on longitudinal or transverse sections of a black rice seed. A–E, Longitudinal section images. Scale bar: 1.0 mm. F–J, Transverse section images. Upper side in transverse sections is the dorsal side of the black rice seed. Scale bar: 0.5 mm. A, F, Optical images. B, G, Localization pattern of the fragment ions at *m/z* 287, derived from the precursor ions at *m/z* 449, corresponding to cyanidin-3-*O*-hexoside. C, H, Localization pattern of the fragment ions at *m/z* 287, derived from the precursor ions at *m/z* 419, corresponding to cyanidin-3-*O*-pentoside. D, I, Localization pattern of the fragment ions at *m/z* 317, derived from the precursor ions at *m/z* 449, corresponding to petunidin-3-*O*-pentoside. E, J, Merged ion image of *m/z* 449→287 (red), *m/z* 419→287 (green), and *m/z* 449→317 (blue). K, Four regions defined in the intensity quantification of each fragment ion. L–N, Relative intensity of the fragment ions corresponding to cyanidin-3-*O*-hexoside (L), cyanidin-3-*O*-pentoside (M), and petunidin-3-*O*-pentoside (N). AV: anterior ventral region, PV: posterior ventral region, PD: posterior dorsal region, AD: anterior dorsal region. Data was collected from 7 sections of 3 black rice seeds (means ± S.E.). Different denote significant differences among groups means from a Tukey-Kramer test (*p*<0.01). O, HPLC chromatogram of anthocyanins in the black rice crude extract. Peak 1 and 2, unknown; peak 3, cyanidin-3-*O*-glucoside; peak 4, peonidin-3-*O*-glucoside. P, Q, Contents of cyanidin-3-*O*-glucoside (P) and peonidin-3-*O*-glucoside (Q) in each part of black rice seeds determined by HPLC and expressed in nmol per mg of seed pieces ± S.E in triplicate.

To test the hypothesis, we examined the localization of 2 anthocyanin species that had the same aglycone but a different sugar moiety. In this experiment, we compared the distributions of cyanidin-3-*O*-hexoside (*m/z* 449→287), cyanidin-3-*O*-pentoside (*m/z* 419→287), and petunidin-3-*O*-pentoside (*m/z* 449→317). As mentioned above, the product ions derived from the precursor ions of cyanidin-3-*O*-hexoside (*m/z* 449→287) were localized in the posterior dorsal side of the pericarp (Fig. 5B); the fragment ions derived from the precursor ions of cyanidin-3-*O*-pentoside (*m/z* 419→287) and petunidin-3-*O*-pentoside (*m/z* 449→317) were distributed in the entire pericarp (Fig. 5C, D). Their merged image is illustrated in Figure 5E. We analyzed a transverse section of black rice to confirm the localization pattern of these anthocyanins. Cyanidin-3-*O*-hexoside (*m/z* 449→287) was localized predominantly in the dorsal side (Fig. 5G); cyanidin-3-*O*-pentoside (*m/z* 419→287) was colocalized with petunidin-3-*O*-pentoside (*m/z* 449→317) over a broad region of the pericarp (Fig. 5H, I). Their merged image is depicted in Figure 5J. Next, we quantified the intensity of each fragment ion in 4 regions on longitudinal sections of black rice (Fig. 5K). The intensity of the fragment ions corresponding to cyanidin-3-*O*-hexoside (*m/z* 449→287) was significantly higher in the posterior dorsal region than in the other regions (*p*<0.01, Fig. 5L). The intensity of the fragment ions corresponding to cyanidin-3-*O*-pentoside (*m/z* 419→287) (Fig. 5M) and to petunidin-3-*O*-pentoside (*m/z* 449→317) (Fig. 5N) was higher in the anterior dorsal region than in the other regions. Quantification of each fragment ion revealed the different localization patterns of anthocyanins in the pericarp of black rice. To confirm the results of quantitative analysis of MALDI-IMS/MS, we quantitated the contents of anthocyanin of each seed parts by an independent quantitative method. The black rice seed was divided into 4 pieces as mentioned in Figure 5K. Then anthocyanins in each part of 30 seeds were extracted, followed by separation and detection by high performance liquid chromatography (HPLC). HPLC chromatogram of anthocyanins from whole black rice seeds is shown in Fig. 5O. According to the retention time with corresponding authentic standards, peaks 3 and 4 were identified as cyanidin-3-*O*-glucoside and peonidin-3-*O*-glucoside, respectively. Peaks 1 and 2 could not be identified by comparison with authentic standards. Further studies will be needed to identify molecular species of these compounds. Although the peaks corresponded to cyanidin-3-*O*-pentoside and petunidin-3-*O*-pentoside were not detected in our HPLC analysis, cyanidin-3-*O*-glucoside and peonidin-3-*O*-glucoside in each seed part were successfully identified and quantified (Fig. 5P, Q). The contents of anthocyanin species quantified by HPLC analysis were in good agreement with the results of quantitative analysis of MALDI-IMS/MS (Fig. 5L and data not shown). These results indicate that the quantification of ion intensity on MALDI-IMS/MS image reflects practically the contents of anthocyanin in black rice seeds. Although the biological consequences of different distribution patterns of anthocyanin molecular species in the pericarp of black rice seed are not clearly defined now, our data suggested that anthocyanin species composed of different sugar moieties have specifically-different biological functions in black rice seed.

## Discussion

Although the distribution and composition of anthocyanin species in various plants have been studied previously [29], information about the interstitial localization of anthocyanin species in plant tissue is limited. In this study, we revealed the different localization patterns of anthocyanin species in black rice and demonstrated the efficacy of MALDI-IMS analysis for the detection, identification, and mapping of the various anthocyanin species in plant tissue.

Anthocyanins, a group of reddish-purple flavonoids, are the primary pigments in red and black seeds, and are responsible for the attractive red, purple, and blue colors of many flowers, fruits, and vegetables. Among the many species of naturally occurring anthocyanins, cyanidin-3-*O*-glucoside is the most common in higher plants [29]. Cyanidin-3-*O*-glucoside has been recognized as a potent bioactive molecule that protects against TNF-α–induced endothelial dysfunction [30], ethanol neurotoxicity in the developing brain [31], and tumor promoter-induced carcinogenesis and tumor metastasis [32] because of its high antioxidative activity. In addition, cyanidin-3-*O*-glucoside improves triglyceride metabolism in the liver of diabetic KK-*Ay* mice by inhibiting mitochondrial glycerol-sn-3-phosphate acyltransferase 1 activity through the phosphorylation of protein kinase C zeta [33]. Previous studies on the identification and quantification of anthocyanins in black rice have shown cyanidin-3-*O*-glucoside as the primary anthocyanin species [13], [14], [15], [16]. The data from this study support these previous studies (Figs. 1D, 5O). MALDI-MS and MS/MS analysis of anthocyanin-rich extract from black rice identified 7 species of anthocyanin monoglycosides and 2 species of anthocyanin diglycosides (Fig. 2, Table 1). MALDI-IMS and MS/MS analysis on tissue sections revealed the existence of an additional 2 species of anthocyanin pentoside in the pericarp of black rice (Fig. 4A, B). These results suggest that MALDI-IMS analysis of tissue sections is more effective for identifying anthocyanins than MALDI-MS analysis of black rice crude extracts using acidified methanol as a solvent. Acidified methanol is an effective solvent for extracting anthocyanins from various plants (blueberry, potato, red cabbage); the acid lowers pH levels and prevents the degradation of anthocyanins. However, because hydrochloric or formic acid becomes concentrated as the acidified methanol evaporates, anthocyanin monoglycosides are converted into unstable aglycones [29]. Therefore, the minor anthocyanins in the black rice crude extracts may be degraded into sugar and aglycones during the evaporation step, leading to the trace detection of 2 species of anthocyanin pentosides in the crude extracts by MALDI-MS analysis (Fig. 2C, D). Even in the crude extracts prepared without concentration step, the minor anthocyanins were not identical in both MALDI-MS/MS and HPLC analysis probably due to their low amounts or less stability in extracting solvents (Figs. 2C, 2D, 5O). As a preparation step such as extracting and concentrating the target molecules is not required, MALDI-IMS analysis of tissue sections is a more effective method for identifying and mapping anthocyanin molecular species in plant tissues.

In this study, we conducted a detailed investigation of the localization of anthocyanin species in black rice tissue. Analysis of black rice sections by MALDI-IMS/MS revealed the different localization patterns of anthocyanin species composed of different sugar moieties. From the longitudinal section of black rice, we determined that cyanidin-3-*O*-hexoside (Figs. 4D, 5B) and peonidin-3-*O*-hexoside (Fig. 4I) were localized in the dorsal posterior pericarp. Observations of the transverse section determined that cyanidin-3-*O*-hexoside was localized predominantly in the dorsal pericarp (Fig. 5G). In contrast, cyanidin-3-*O*-pentoside (Fig. 5C), petunidin-3-*O*-pentoside (Figs. 4E, 5D) and malvidin-3-*O*-pentoside (Fig. 4J) were localized over a broad region of the pericarp. Glycosylation of anthocyanidins by glycosyltransferases increases their chemical stability and aqueous solubility in vacuoles. UDP-glucose: flavonoid 3-*O*-glucosyltransferase (UF3GT) is a well-characterized glycosyltransferase that catalyzes the transfer of glucose from UDP-glucose to the 3-position of anthocyanidins to form the corresponding anthocyanins. The expression of the *uf3gt* gene is one of the most regulated steps in anthocyanin synthesis and is required for the accumulation of anthocyanins in some plants [34], [35], [36], [37], [38], [39]. The seeds of the *Arabidopsis thaliana* mutant, *anthocyaninless1* (*ANL1*) have a reduced anthocyanin content, and the gene responsible for *ANL1* encodes UF3GT [36]. The rice genome has 2 orthologues of *uf3gt*, Os06g0192100 and Os07g0148200. Although these 2 orthologues have not been well-characterized, the mechanism of *uf3gt* expression in *A. thaliana* has been characterized. In *A. thaliana* seedlings, *uf3gt* expression is strongly up-regulated by sucrose [40]. In addition, *A. thaliana uf3gt* expression is induced by the *production of anthocyanin pigment 1* MYB transcription factor, which is up-regulated by light exposure [41] and sucrose [42]. Sunlight exposure increases anthocyanin production in black rice [43], and several MYB transcription factors were identified as being seed-specific and regulating anthocyanin biosynthesis [44]. In the developing caryopses of rice, assimilated carbon such as sucrose is transported through the vascular parenchyma and pigment strand in the dorsal region of the rice seed, and forms a concentration gradient between the dorsal and ventral parts of the pericarp [45]. Sunlight and sucrose are thought to coordinate to induce *uf3gt* expression in the dorsal pericarp, resulting in dorsal localization patterns of anthocyanin species composed of a hexose moiety. This study will contribute to the investigation of expression patterns of anthocyanin-related genes and a better understanding of the biological significance of anthocyanin species in black rice.

## Materials and Methods

### Materials

Adhesive film (Cryofilm type IIC [10]) was purchased from Leica Microsystems (Tokyo, Japan). Glass slides (Fisherbrand Superfrost Plus) were purchased from Thermo Fisher Scientific (San Jose, CA, USA). Methanol and distilled water were purchased from Nacalai Tesque (Kyoto, Japan). 2,5-Dihydroxybenzoic acid (DHB) and carboxymethyl cellulose (CMC) were obtained from Bruker Daltonics (Bremen, Germany) and Wako Pure Chemical Industries (Osaka, Japan), respectively. Cyanidin-3-*O*-glucoside and peonidin-3-*O*-glucoside were purchased from Fujicco (Hyogo, Japan) and Tokiwa phytochemical (Chiba, Japan), respectively. All chemicals used in this study were of the highest purity available. Black rice (*Oryza sativa* L.) seeds harvested in Hyogo, Japan, in 2010 (Fig. 1A) were purchased from a local market in Nara, Japan.

### Anthocyanin extraction from black rice

Mortars and pestles were used to grind 20 g of black rice. 1 g of the ground rice samples was extracted in 10 mL of methanol containing 1% HCl (v/v) for 24 hours in the dark at 4°C. The crude extracts were filtered with Whatman No. 2 paper. For quantification of anthocyanins in black rice seed parts, a black rice seed was divided into 4 pieces defined as anterior ventral (AV), posterior ventral (PV), posterior dorsal (PD), and anterior dorsal (AD) by a fine surgical knife as mentioned in Fig. 5K. Weight of each part pieces of 30 black rice seeds were 147 mg (AV), 165 mg (PV), 159 mg (PD), and 153 mg (AD). Then, anthocyanins were extracted from each part pieces of 30 seeds in 300 µL of methanol containing 1% HCl (v/v) for 2 days at 4°C. The crude extracts from seed parts were obtained by centrifugation at 10,000 g for 1 minute to remove debris. These crude extracts were then subjected to HPLC.

### Quantification of anthocyanins in black rice seeds by HPLC

HPLC analysis was performed using a liquid chromatography system (SCL-10A, Shimadzu, Kyoto, Japan) equipped with two pumps (LC-10A), a control system (SCL-10A), a diode array detector (SPD-20A), a CTA-20A column oven, and a column COSMOSIL 5C_18_-AR-II 4.6×250 mm i.d. 5 µm (Nacalai tesque, Kyoto, Japan). The mobile phase consisted of water∶formic acid (96∶4, v/v) (A phase) and methanol∶formic acid (96∶4, v/v) (B phase). Sample elution used the time gradient program: 0–0.01 min 10% B, 0.01–5.00 min 20% B; 5.01–50.00 min 50% B; 50.01–60.00 min 100% B. 10 µl of sample were injected. Quantification was performed at λ = 520 nm. All chromatographic tests were carried out at 37°C with a flow of 0.5 mL/min. Cyanidin-3-*O*-glucoside and peonidin-3-*O*-glucoside was quantified using a calibration curve of the corresponding standard compounds. All determinations were performed in triplicate.

### MALDI-MS and MS/MS analysis of anthocyanins

A 5-µL aliquot of black rice crude extract was mixed with an equal volume of 50 mg/mL DHB in methanol/water (7∶3, v/v). The mixed sample had been deposited onto glass slide and dried before MALDI-MS analysis. MALDI-MS analysis was performed using a MALDI linear quadrupole ion trap mass spectrometer (MALDI LTQ-XL; Thermo Fisher Scientific) equipped with a 337-nm nitrogen laser at a repetition rate of 60 Hz in positive ion mode. The laser energy was set to 8 µJ, and the mass spectrometer was operated in automatic gain control (AGC) mode. The number of micro scan and sweep shot were set to 1. Ions with *m/z* values in the range of 200–550 were measured. For MS/MS analysis, the selected precursor ions and the product ions obtained by collision-induced dissociation were ejected from the ion trap and analyzed. The collision energy was set to 20% of the maximum available energy required to completely fragment the precursor ion formed from the peptide Met-Arg-Phe-Ala [46]. The laser energy was set to 8 µJ. The number of MSn micro scan and sweep shot were set to 1.

### Preparation of black rice sections (Kawamoto method)

Black rice sections were prepared according to the Kawamoto method with slight modifications [27], [47]. Briefly, black rice seeds were freeze-embedded with 2% CMC at −80°C. The frozen black rice seeds were attached to adhesive film and sliced to 10-µm thickness using a cryostat (CM 1850; Leica Microsystems, Wetzler, Germany). Sections were attached to a glass slide with electrically conductive adhesive tapes.

### Imaging mass spectrometry

MALDI-IMS analysis was performed using the LTQ-XL mass spectrometer as described above. Samples were prepared according to the method published previously [27]. Briefly, 50 mg/mL DHB in methanol/water (7∶3, v/v) was used as a matrix. The DHB matrix solution (500 µL) was sprayed uniformly over the black rice sections using an airbrush with a 0.2-mm nozzle (Procon Boy FWA Platinum; Mr. Hobby, Tokyo, Japan). Data were acquired with a 50-µm step size in positive ion mode. The laser energy was set to 30 µJ, and the mass spectrometer was operated in AGC mode. The number of micro scan and sweep shot were set to 1. Ions with *m/z* values in the range of 350–1000 were measured. ImageQuest software (Thermo Fisher Scientific) was used to create 2-dimensional ion -density maps, normalize peak intensity, and to adjust the color scale. For MS/MS analysis of the anthocyanins in the sections, the collision energy was set to 35% of the maximum available energy for the LTQ-XL, and the laser energy was set to 30 µJ. The number of MSn micro scan and sweep shot were set to 2 and 5, respectively. Following MS/MS analysis, the same section was analyzed by MS as mentioned above or by another MS/MS. After MALDI-IMS analysis, the sections were stained with toluidine blue O. Semi-quantitative analyses of the ion intensity in a part of black rice section were carried out using ImageQuest software. Data was collected from 7 sections of 3 black rice seeds. Multiple comparisons between groups were made by Tukey-Kramer test.
